# Retraction: Long Non-coding RNA LINC00320 Inhibits Tumorigenicity of Glioma Cells and Angiogenesis Through Downregulation of NFKB1-Mediated AQP9

**DOI:** 10.3389/fncel.2022.858255

**Published:** 2022-02-08

**Authors:** Frontiers Editorial Office

The journal and Chief Editors retract the 22 December 2020 article cited above.

Following publication, concerns were raised regarding the validity of the data in the article. The authors failed to provide a satisfactory explanation during the investigation, which was conducted in accordance with Frontiers' policies. Given the concerns, the editors no longer have confidence in the findings presented in the article.

This retraction was approved by the Chief Editors of Frontiers in Cellular Neuroscience and the Chief Executive Editor of Frontiers. The authors agreed with this retraction.

